# Comparative Early Postoperative Outcomes in Acute Calculous vs. Acute Acalculous Cholecystitis: A Retrospective Analysis

**DOI:** 10.3390/medicina62050834

**Published:** 2026-04-27

**Authors:** Jakub Włodarczyk, Wojciech Czernik, Aleksandra Osielczak, Kasper Maryńczak, Arkadiusz Jakubowski, Marcin Włodarczyk, Łukasz Dziki

**Affiliations:** Department of General and Oncological Surgery, Medical University of Lodz, 92-213 Lodz, Poland; wojciech.czernik@stud.umed.lodz.pl (W.C.); aleksandra.osielczak@stud.umed.lodz.pl (A.O.); kasper.marynczak@umed.lodz.pl (K.M.); arkadiusz.jakubowski@stud.umed.lodz.pl (A.J.); marcin.wlodarczyk@umed.lodz.pl (M.W.); lukasz.dziki@umed.lodz.pl (Ł.D.)

**Keywords:** acute cholecystitis, acalculous cholecystitis, calculous cholecystitis, cholecystectomy, postoperative complications, Clavien–Dindo, comprehensive complication index, mortality

## Abstract

*Background and Objectives:* Acute cholecystitis is a common indication for emergency surgery. While acute calculous cholecystitis (ACC) is most common, acute acalculous cholecystitis (AAC) occurs without gallstones and is often associated with severe systemic illness. We compared early postoperative outcomes after cholecystectomy for AAC versus ACC, with emphasis on complication severity and overall morbidity burden. *Materials and Methods:* We performed a single-center retrospective cohort study of consecutive adults undergoing urgent or emergent cholecystectomy for acute cholecystitis between December 2020 and April 2025. Patients with chronic cholecystitis, duplicate records, missing group assignment, or incomplete 30-day follow-up were excluded. The primary 30-day endpoints were postoperative complications, their severity (assessed with Clavien–Dindo scale), and cumulative morbidity assessed using the Comprehensive Complication Index. Secondary outcomes included operative approach, postoperative length of stay, 30-day readmission, and mortality. *Results:* A total of 221 patients were analyzed (181 ACC, 40 AAC). Patients with AAC were older and more frequently male. Any complication within 30 days occurred substantially more often in AAC patients than in ACC patients. Morbidity severity also differed markedly, with higher-grade complications occurring more frequently in the AAC group. AAC patients exhibited a substantially greater overall morbidity burden, indicating not only more frequent complications but also a heavier cumulative impact. Thirty-day mortality was considerably higher in AAC. Open surgery was more commonly required in AAC, whereas postoperative length of stay and 30-day readmission rates were similar between groups. *Conclusions:* In this cohort, AAC was associated with substantially worse early outcomes after cholecystectomy than ACC, characterized by a pronounced increase in clinically significant complications (Clavien–Dindo ≥ IIIa), greater cumulative morbidity (CCI), and markedly higher 30-day mortality. These findings support treating AAC as a high-risk phenotype warranting intensified perioperative optimization and vigilant postoperative monitoring.

## 1. Introduction

Acute cholecystitis is a common urgent surgical condition and a frequent reason for emergency cholecystectomy [1,2]. Most cases arise from cystic duct obstruction due to gallstones—acute calculous cholecystitis. In contrast, acute acalculous cholecystitis occurs without gallstone-related cystic duct obstruction and is often associated with severe systemic illness [3,4]. Proposed mechanisms in acalculous disease include hypoperfusion, ischemia, cholestasis, and microvascular injury [3]. These are linked to higher risks of gangrene, perforation, and sepsis [4].

Worldwide, early laparoscopic cholecystectomy is the guideline-recommended standard treatment for acute calculous cholecystitis [2,5,6,7]. Eurostat’s 2022 “Surgical operations and procedures” statistics show that cholecystectomy rates vary across EU member states (e.g., 257.9 per 100,000 inhabitants in Lithuania; 103.6 per 100,000 inhabitants in Bulgaria; 2022 data) and that the laparoscopic approach predominates, with laparoscopic shares ranging from 79% (Bulgaria) to 96% (Netherlands, 2021 data) [8]. Population-based cohort evidence from England further supports index-admission emergency cholecystectomy as a standard approach associated with improved outcomes in acute cholecystitis [9].

Overall, postoperative morbidity after cholecystectomy for acute cholecystitis remains clinically meaningful—particularly in older and patients with comorbidities—reinforcing the need for robust comparative outcome data and standardized reporting [5,6,9]. Despite extensive clinical experience, contemporary comparative outcome data for acalculous versus calculous cholecystitis remain limited and heterogeneous. Standardized outcome tools—the Clavien–Dindo Classification for complication severity and the Comprehensive Complication Index (CCI) for cumulative morbidity—facilitate consistent comparison of early postoperative risk profiles and can inform operative planning and postoperative surveillance [10,11,12].

Against this background, we aimed to compare early postoperative outcomes between acute calculous and acute acalculous cholecystitis to determine whether acalculous disease constitutes a clinically distinct high-risk phenotype.

## 2. Materials and Methods

This single-center retrospective cohort included all adults who consecutively underwent urgent or emergent cholecystectomy for acute cholecystitis between December 2020 and April 2025. Diagnoses were corroborated clinically, radiologically, intraoperatively, and histopathologically. Exclusion criteria included duplicate entries, missing group assignment, or missing postoperative outcome assessment. Patients managed nonoperatively, including those treated with percutaneous cholecystostomy without cholecystectomy during the index admission, were not included in the analytic cohort. Chronic cholecystitis was also excluded. Cases were classified as calculous cholecystitis when cystic duct obstruction by a gallstone was identified on ultrasonography, intraoperative assessment, and/or histopathology. Cases were classified as acalculous cholecystitis when acute cholecystitis was present but no obstructing cystic duct stone was identified through the available ultrasonographic, intraoperative, and histopathologic assessments. Microlithiasis was classified as calculous disease. Cases with biliary sludge without an obstructing stone were excluded. Patients with cholelithiasis without acute cholecystitis were also excluded.

The final analytic sample comprised 181 patients with calculous cholecystitis and 40 with acalculous cholecystitis. Decisions for surgery reflected guideline-directed care favoring early cholecystectomy where feasible [2,5,6,7], consistent with population-based cohort evidence supporting index-admission emergency cholecystectomy [9].

Baseline variables included age, sex, and American Society of Anesthesiologists (ASA) Physical Status [13]. Charlson Comorbidity Index (Charlson CI) score [14], presenting symptoms, preoperative antithrombotic therapy by class, duration of symptoms, and time from admission to surgery were also recorded. Time from admission to surgery was recorded as the number of days from hospital admission to operation, regardless of whether the index admission was initially for surgical reasons (0 = same-day surgery, 1 = next-day surgery, 2 = surgery 2 days after admission, etc.). Because some patients, particularly in the acalculous group, developed cholecystitis during an ongoing non-surgical hospitalization, this variable reflects elapsed time from hospital admission rather than time from surgical qualification or onset of biliary symptoms. For descriptive interpretation, time from admission to surgery was also examined categorically as same-day, next-day, and ≥2 days after admission; this information is presented in Table 1. Operative variables included the operative approach (laparoscopic, open, conversion) and intraoperative complications. For descriptive characterization of the acalculous cohort, additional chart-level variables were retrieved, including ICU admission before surgery, ICU total stay, sepsis, vasopressor requirement, recent major surgery, prolonged fasting, total parenteral nutrition, mechanical ventilation, major cardiovascular events or cardiovascular surgery, and other severe systemic illnesses.

The primary outcome was postoperative morbidity assessed in a prespecified three-part framework: (i) occurrence of any postoperative complication within 30 days of the index operation; (ii) complication severity using the Clavien–Dindo Classification (full orginal distribution and a prespecified ≥ IIIa vs. 0–II contrast) [10,11]; and (iii) cumulative morbidity summarized by the Comprehensive Complication Index (CCI) [12]. Secondary outcomes were length of stay after surgery (LOS), 30-day readmission, and 30-day mortality. Complications were identified from the electronic health records and graded independently by two investigators using the Clavien–Dindo classification; disagreements were resolved by consensus. In addition to Clavien–Dindo classification and the Comprehensive Complication Index, postoperative complications occurring within 30 days were grouped into clinically relevant categories for descriptive reporting in Table 2. All postoperative complications occurring within 30 days were included in the CCI calculation. Complications were first graded independently using the Clavien–Dindo classification, after which the CCI was derived for each patient. When multiple complications occurred in a single patient, all were entered into the calculation. Death corresponded to a CCI of 100. CCI values were obtained using the published online CCI calculator (https://www.cci-calculator.com/cci-calculator (accessed on 16 January 2026)). Postoperative complications and other thirty-day outcomes were ascertained from the electronic health record and supplemented by telemonitoring and outpatient follow-up review where applicable. Postoperative infectious complications were classified and treated according to current guidelines for the management of intra-abdominal infections [15].

Preoperative inflammatory markers were defined a priori at admission and reassessed on the day of surgery. Markers included C-reactive protein (CRP), white blood cell count (WBC), neutrophil-to-lymphocyte ratio (NLR), and platelet-to-lymphocyte ratio (PLR). The respective values are shown in Table S1.

Between-group comparisons used Pearson’s χ^2^ (or Fisher’s exact when expected counts were below 5) with odds ratios and 95% confidence intervals for categorical variables. Continuous variables were analyzed using a *t*-test or Mann–Whitney U as appropriate. Spearman’s ρ and the Kruskal–Wallis test were applied to Clavien–Dindo grades, and Wilcoxon/Mann–Whitney U tests were used for two-group ordinal contrasts, with a prespecified contrast of 0–II vs. ≥IIIa for interpretability. Continuous variables are reported as mean ± standard deviation or median [interquartile range]. Categorical variables are reported as counts with percentages. Effect directions were standardized: odds ratios compare calculous with the acalculous condition (OR < 1 indicates lower odds in calculous patients). Differences for continuous endpoints are reported as acalculous minus calculous (Δ > 0 means higher values in the acalculous group). Statistical analyses were performed in R (version 4.5.1; R Foundation for Statistical Computing, Vienna, Austria) [16] using RStudio (version 2025.09; Posit Software, Boston, MA, USA) [17]. Continuous and categorical summaries were generated using base R and dplyr. To account for the substantial baseline differences between groups, four prespecified multivariable analyses were performed. Covariates common to all models were: acalculous etiology (AAC vs. ACC as reference), age, sex, ASA Physical Status, Charlson Comorbidity Index, and time from admission to surgery (as a continuous variable). Operative approach (open vs. laparoscopic as reference; conversion as a separate category) was additionally included as a covariate in models where it was not itself an outcome of interest. Binary logistic regression was used to model any postoperative complication within 30 days (*n* = 221; 66 events) and severe complications (Clavien–Dindo grade ≥ IIIa; *n* = 221; 44 events). For the any-complication model, all six common covariates and operative approaches were included (eight predictors; events-per-variable ratio 8.2). The severe-complication model retained the six common covariates without operative approach (six predictors; events-per-variable ratio 7.3). For 30-day mortality (*n* = 221; 23 events), a parsimonious model was used, retaining only acalculous etiology, age, and ASA Physical Status as covariates (events-per-variable ratio 7.7), to avoid model overfitting given the limited event count. The results of the logistic models are reported as odds ratios with 95% confidence intervals. The Comprehensive Complication Index was modeled as a continuous outcome using ordinary least-squares linear regression with the same predictor set as the any-complication model; results are reported as regression coefficients (β) with 95% confidence intervals. The contribution of antithrombotic therapy to any postoperative complication was evaluated in a sensitivity analysis by adding it as an additional covariate to the any-complication model; since neither the likelihood-ratio test (*p* = 0.137) nor the Akaike Information Criterion supported its inclusion, it was omitted from all primary models. Postoperative length of stay was reported as a median with interquartile range, given the right-skewed distribution inherent to such data, and compared using the Mann–Whitney U test.

The study was conducted as a retrospective analysis of existing medical records only. No additional diagnostic or therapeutic procedures were performed, no intervention was introduced, and no contact with patients occurred for the purposes of this research. Accordingly, this study does not meet the criteria of a medical experiment within the meaning of the Polish Act of 5 December 1996 on the professions of physician and dentist (Journal of Laws 2023, item 1516, as amended), which defines a medical experiment as an interventional procedure performed on a human subject. This legal interpretation was reviewed and confirmed by the Bioethics Committee of the Medical University of Łódź, which stated that the present study does not constitute a medical experiment and therefore does not require approval as an interventional study. All data were processed in compliance with applicable data protection and ethical standards.

## 3. Results

We analyzed 221 adults (181 calculous, 40 acalculous). Acalculous patients were older (72.2 ± 11.5 vs. 61.9 ± 16.0 years) and more often male (30/40 [75.0%] vs. 85/181 [47.0%]). Baseline characteristics are summarized in Table 3. Comorbidity and perioperative risk were higher in the acalculous group, with a higher Charlson Comorbidity Index (median 5.0 vs. 3.0) and a higher ASA Physical Status distribution (more ASA III-V). Blood thinner use was also more frequent in the acalculous cohort. An additional chart review showed that the acalculous cohort frequently presented in the context of broader systemic illness: 3/40 patients had ICU admission before surgery, 3/40 had sepsis, 13/40 required vasopressors, 7/40 had recent major surgery, 2/40 had prolonged fasting, 1/40 had preoperative total parenteral nutrition, 9/40 required mechanical ventilation, and at least 29/40 had a major cardiovascular event or prior cardiovascular surgery; other severe systemic illnesses were documented in 5/40 patients. Moreover, 3/40 (7.5%) patients had ICU admission before surgery, and among those with documented ICU stay, the total ICU stay was 3.0 days [2.0–6.0], while the three patients admitted to ICU before surgery had a median ICU stay of 6.0 days [5.0–7.5]. The presenting symptoms and timing variables are shown in Table 1.

To address the substantial baseline differences between groups, we performed four adjusted analyses. The covariates included in all models were: acalculous etiology (AAC vs. ACC), age, sex, ASA Physical Status, Charlson Comorbidity Index, and time from admission to surgery. The operative approach (open vs. laparoscopic as reference; conversion as a separate category) was also included in models where it was not itself an outcome of interest.

In a multivariable binary logistic regression for any postoperative complication (*n* = 221; 66 events), acalculous etiology remained an independent predictor of morbidity after adjustment for all covariates (OR 2.76, 95% CI 1.16–6.54; *p* = 0.021). No other covariate reached statistical significance in this model, including operative approach (open vs. laparoscopic: OR 1.67, 95% CI 0.74–3.75; *p* = 0.214) and age (OR 1.03 per year, 95% CI 1.00–1.07; *p* = 0.079). The model’s pseudo-R^2^ was 0.213.

For severe complications (Clavien–Dindo grade ≥ IIIa; *n* = 221; 44 events), acalculous etiology showed a consistent, though statistically non-significant, association with higher odds of severe complications (OR 2.30, 95% CI 0.96–5.55; *p* = 0.063). Age was the only independent predictor reaching significance (OR 1.05 per year, 95% CI 1.01–1.09; *p* = 0.012).

For 30-day mortality (*n* = 221; 23 events), a parsimonious logistic regression was applied to avoid model overfitting, retaining acalculous etiology, age, and ASA Physical Status as covariates. Acalculous etiology was an independent predictor of 30-day mortality (OR 4.08, 95% CI 1.49–11.16; *p* = 0.006). Older age was also independently associated with mortality (OR 1.07 per year, 95% CI 1.02–1.12; *p* = 0.003), while ASA score showed a trend that did not reach significance (OR 1.90, 95% CI 0.97–3.71; *p* = 0.059).

Finally, a linear regression for the Comprehensive Complication Index confirmed that acalculous etiology was independently associated with a significantly higher cumulative morbidity burden (β = +18.45 points, 95% CI 7.93–28.98; *p* = 0.001), adjusting for all baseline covariates and operative approach (R^2^ = 0.250). The results of all of the multivariable analyses are presented in Table 4.

**Figure 1:**
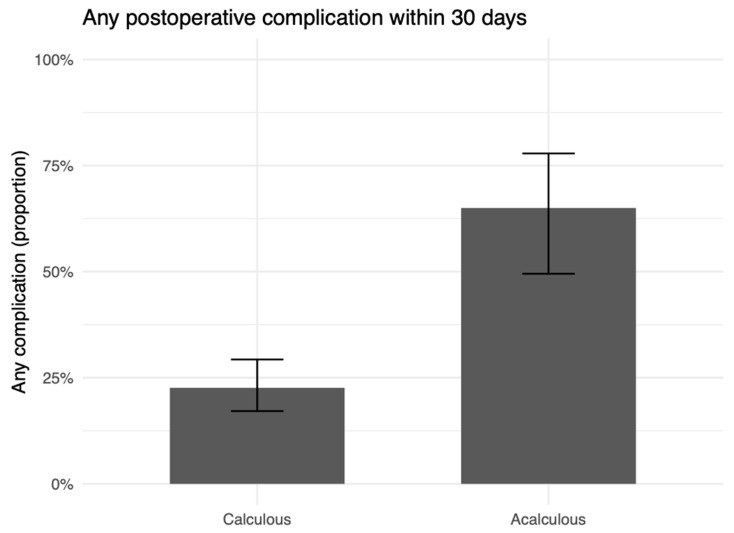
Any postoperative complication within 30 days by etiology. Bars show group proportions with 95% CIs.

**Figure 2:**
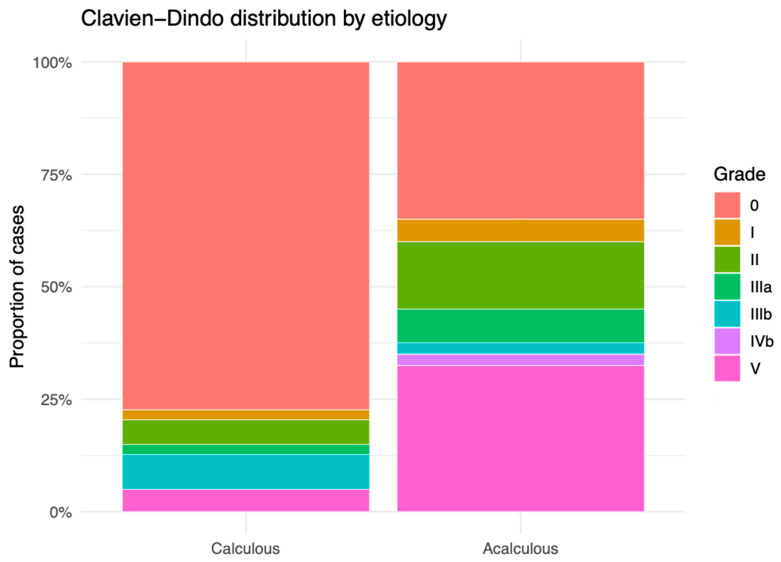
Clavien–Dindo distribution by etiology. Bars show within-group grade proportions. Severe complications (≥IIIa) occurred in 18/40 (45.0%) acalculous vs. 27/181 (14.9%) calculous patients. OR (calculous vs. acalculous) = 0.22 (95% CI 0.10–0.49); *p* < 0.001. Overall ordinal shift across grades: *p* < 0.001.

**Figure 3:**
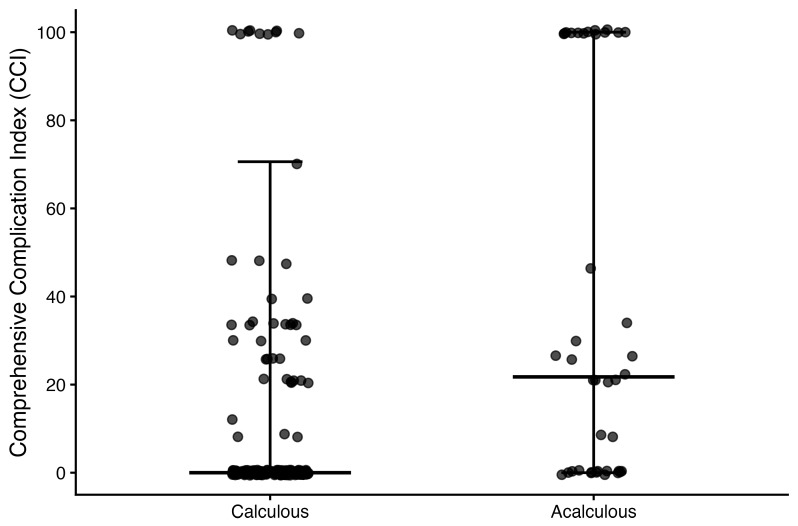
Comprehensive Complication Index (CCI) by etiology. Points represent individual observations. The horizontal line indicates the median and whiskers indicate the 5–95th percentiles. Mean difference (acalculous − calculous) Δ = 30.1 (95% CI 15.8–44.4); *p* < 0.001.

Postoperative morbidity was consistently worse in the acalculous group. Any complication within 30 days occurred in 26/40 (65.0%) acalculous cases versus 41/181 (22.7%) calculous cases (OR 0.16, 95% CI 0.07–0.35; *p* < 0.001; Figure 1; Table 5). Severe complications (Clavien–Dindo ≥ IIIa) occurred in 18/40 (45.0%) versus 27/181 (14.9%) (OR 0.22, 95% CI 0.10–0.49; *p* < 0.001; Figure 2; Table 5). Cumulative morbidity was higher in acalculous cases, with a mean CCI difference (acalculous − calculous) of 30.1 (95% CI 15.8–44.4; *p* < 0.001; Figure 3; Table 5).

**Figure 4:**
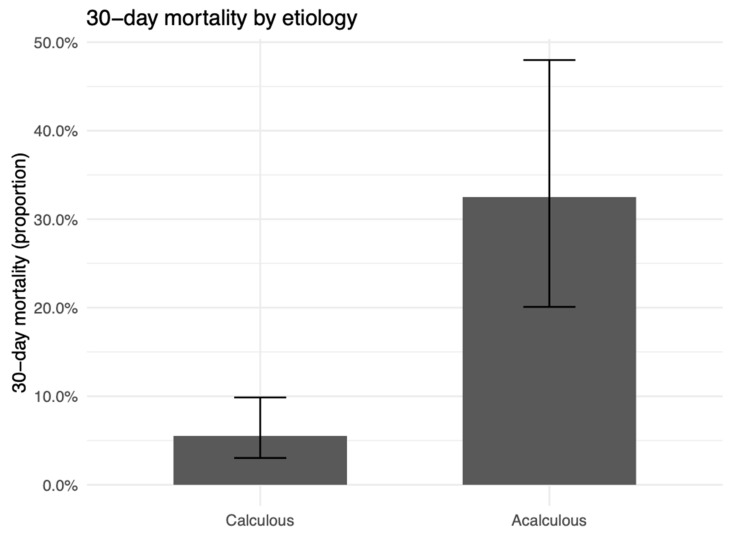
Thirty-day mortality by etiology. Bars show group proportions with 95% CIs. Acalculous: 13/40 (32.5%); calculous: 10/181 (5.5%). OR (calculous vs. acalculous) = 0.12 (95% CI 0.04–0.34); *p* < 0.001.

Thirty-day mortality was higher in the acalculous group (13/40 [32.5%] vs. 10/181 [5.5%]; OR 0.12, 95% CI 0.04–0.34; *p* < 0.001; Figure 4; Table 5). Among the 13 deaths in the acalculous group, chart-documented events were predominantly circulatory collapse or cardiac arrest, usually asystole, often in the setting of cardiogenic or mixed septic–cardiogenic shock and/or multiorgan failure; one death followed respiratory arrest with pulseless electrical activity, and one had an undocumented mechanism. The operative approach differed, with open surgery being more common in acalculous cases (32/40 [80.0%] vs. 80/181 [44.2%]) (Table 5). The thirty-day readmission (4/40 [10.0%] vs. 8/181 [4.4%]) and postoperative length of stay (median [IQR]: ACC 3 [2–5], AAC 4.5 [2–7] days) were similar between groups (Table 5). The specific postoperative complication types observed in each group are detailed in Table 2.

Inflammatory markers are summarized in Table S1 (adjusted *p*-values). CRP and NLR were higher in the acalculous group at admission and on the day of surgery. WBC did not differ after adjustment at either timepoint, while PLR was higher in acalculous cases on the day of surgery but not at admission (Table S1).

## 4. Discussion

This study delineates acalculous cholecystitis as a substantially higher-risk phenotype in comparison to calculous cholecystitis: patients were older with greater comorbidity, operations more often required an open approach, and postoperative outcomes showed a marked shift toward severe complications (≥IIIa) with high early mortality. Available chart-level descriptions suggest that this excess mortality was driven mainly by systemic decompensation—most often circulatory collapse/asystolic cardiac arrest in the context of cardiogenic or mixed shock and multiorgan failure—rather than by a single local technical complication.

The observed differences persisted despite standard surgical care and are consistent with current reviews of acalculous etiology in systemically compromised patients, wherein hypoperfusion, ischemia, and cholestasis contribute to gangrene, perforation, and sepsis [3,4]. Contemporary overviews likewise emphasize that acalculous disease disproportionately affects multimorbid and critically ill adults, consistent with our cohort demographic and outcome gradients [6]. Our internal gradients—age, Charlson CI, and LOS increasing with Clavien–Dindo grade—support the construct validity of the severity phenotype and align with broad experience using Clavien–Dindo classification to benchmark surgical outcomes [10,11,18]. The Comprehensive Complication Index (CCI) further quantifies cumulative morbidity beyond the single highest grade [12].

Current guidelines and contemporary reviews stress that, even in high-risk patients, early cholecystectomy should remain first-line when feasible, reserving percutaneous drainage for those unfit for surgery rather than merely “high-risk” [2,5,6,7]. Large population-based and age-specific studies have reported increasing complication rates and healthcare use with advancing age and comorbidity in acute cholecystitis [19,20]. These findings are consistent with the risk profile and early outcomes of our cohort. Some reports suggest that age alone is insufficient once comorbidity is taken into account [21]. Beyond the Tokyo severity [2], risk-estimation efforts continue to evolve for calculous disease [7]. The Chole-Risk score predicts postoperative complications after early laparoscopic cholecystectomy for acute cholecystitis [22], and pragmatic multicenter tools are being prospectively examined [23]. Comparative-effectiveness research in older, multimorbid adults suggests that, where physiologically possible, definitive surgery results in fewer readmissions or ED visits, as well as lower costs, compared to nonoperative care [24]. Studies comparing percutaneous cholecystostomy with cholecystectomy in high-risk patients suggest using drainage primarily as a bridge for those temporarily unfit for anesthesia, while proceeding to definitive cholecystectomy once feasible [25,26]. Comparative evidence also suggests that drainage-first strategies may carry a higher cumulative burden of adverse events when compared with upfront definitive surgery in patients who can physiologically tolerate an operation. In the CHOCOLATE randomized trial of high-risk acute calculous cholecystitis, laparoscopic cholecystectomy resulted in markedly fewer major complications and reinterventions than percutaneous catheter drainage [27]. When drainage is used as a bridge to surgery, outcomes at the eventual cholecystectomy admission may be comparable in selected severe cases, supporting its role primarily for those temporarily unfit for anesthesia or with organ dysfunction [28]. Moreover, in line with contemporary guidance [2,5,6,7] and population-based cohort evidence [9], acalculous patients carry a higher perioperative risk and thus warrant early involvement of a senior attending surgeon, bailout strategies (including subtotal cholecystectomy when Calot’s triangle is unsafe to proceed) [29], rigorous perioperative optimization, and closer postoperative surveillance. These measures adhere to established principles of source control and antimicrobial management in the context of intra-abdominal infections [15].

The higher open approach rate likely reflects the greater baseline frailty and operative complexity of acalculous presentations. In U.S. nationwide data, conversion from laparoscopic to open cholecystectomy occurs in a measurable minority of cases and is associated with identifiable risk factors, supporting conversion/open surgery as an accepted safety strategy in difficult cases [30]. Contemporary Polish real-world registry data similarly provide local benchmarks for operative approach: in the Polish Gallstone Surgery Registry (sex-stratified analysis), open cholecystectomy occurred in 11% of men and 5% of women, and conversion in 5.5% vs. 2.8%, respectively [31]. Notably, this analysis does not differentiate between calculous and acalculous cases. The higher prevalence of antithrombotic therapy in our acalculous cohort may contribute to an “open-prone” risk profile: a recent meta-analysis found that antithrombotic therapy during laparoscopic cholecystectomy is associated with higher risks of intraoperative and postoperative bleeding events and increased transfusion requirements [32]. In acute cholecystitis specifically, emergent cholecystectomy has been reported as feasible in patients receiving antithrombotics, but bleeding-related perioperative considerations remain clinically relevant and may influence operative strategy in high-risk patients [33].

The observed severity shift in the acalculous cohort may reflect cardiovascular hypoperfusion and cholestasis exacerbating local gallbladder injury in already compromised hosts. Observational series describe acute cholecystitis after major cardiovascular surgery [34] and in other hemodynamically vulnerable states [4]. The higher prevalence of antithrombotic therapy and higher ASA scores/Charlson CI in this group may further challenge intra- and postoperative management, offering a pathway to the higher CCI and mortality we observed. Notably, we observed a single grade IVb case and no IVa events, with a pronounced positive skew in grade V among acalculous cases—underscoring why binary “any complication” assessment obscures clinically significant severity differences, whereas the ordinal Clavien–Dindo distribution and CCI reveal the gradient of harm [10,11,12,18].

Key strengths of our study include the rigorous, multidisciplinary consolidation of diagnoses across clinical, radiological, operative, anesthetic, and histopathological domains; the use of a consecutive patient cohort within a defined timeframe; a clearly prespecified statistical analysis plan incorporating both ordinal and binary severity metrics, appropriate statistical tests, multiplicity control, and transparent reporting of effect sizes with corresponding confidence intervals. The use of standardized ordinal and binary severity metrics (Clavien–Dindo and CCI) further strengthens the interpretability [10,11,12,18].

The study has several limitations which merit consideration. The retrospective, single-center design introduces the potential for selection and information biases. The uneven group sizes, while reflective of real-world case distribution, limit statistical power and external validity. In addition, retrospective etiologic classification may not have fully eliminated heterogeneity within the acalculous group, although group allocation was based on ultrasonographic, intraoperative, and histopathologic assessment, and predefined classification rules described in the Materials and Methods were applied to reduce misclassification. Furthermore, formal Tokyo Guidelines severity grading was not recorded, so we could not adjust for guideline-defined cholecystitis severity as a potential contributor to operative selection and postoperative outcomes. Residual confounding may arise from unmeasured variables, such as frailty, specific cardiac function metrics, or nuanced clinical thresholds for escalating care. These factors may influence both the likelihood of acalculous presentation and postoperative outcomes independent of disease etiology. Finally, because the cohort was restricted to patients who underwent cholecystectomy, the findings should not be extrapolated to all patients with acute cholecystitis, particularly those who were initially managed with percutaneous drainage or nonoperative treatment.

Future multisite research should assess whether acalculous disease independently predicts severe complications after adjusting for age, ASA Physical Status, Charlson Comorbidity Index, and operative approach, preferably with propensity or doubly robust methods [35]. Incorporating inflammatory indices may refine preoperative stratification [36]. In our cohort, CRP and NLR were higher in the acalculous group at both admission and on the day of surgery—a pattern consistent with systemic compromise and local ischemic–inflammatory injury [3,4,6]. Practical evaluations of standardized perioperative pathways tailored to this phenotype (senior-first operating, bailout protocols, antithrombotic management, and postoperative escalation criteria) could determine whether coordinated care reduces the observed excess risk. Given the mortality signal, targeted postoperative surveillance and early-warning protocols in this subgroup warrant evaluation.

## 5. Conclusions

This study demonstrates that acute acalculous cholecystitis (AAC) constitutes a clinically distinct high-risk phenotype among patients undergoing urgent or emergent cholecystectomy. Acalculous etiology was an independent predictor of postoperative morbidity, 30-day mortality, and cumulative complication burden after adjustment for age, comorbidity, anesthetic risk, time to surgery, and operative approach—confirming that the excess risk is not solely attributable to the more vulnerable baseline profile of affected patients.

These findings have direct clinical implications. Patients presenting with AAC may benefit from intensified perioperative monitoring, earlier surgical intervention where feasible, and systematic risk stratification using standardized tools such as ASA Physical Status and the Charlson Comorbidity Index. Prospective multicenter studies with larger AAC cohorts are warranted to validate these findings and to identify optimal management strategies for this high-risk population.

## Figures and Tables

**Table 1:** Symptoms characteristics. Values are presented as n/N (%) or mean ± SD. Abbreviations: SD—standard deviation.

Variable	Calculous (*n* = 181)	Acalculous (*n* = 40)	*p*-Value
Pain, n/N (%)	179/181 (98.9%)	37/40 (92.5%)	0.296
Fever, n/N (%)	25/181 (13.8%)	5/40 (12.5%)	1.000
Nausea/vomiting, n/N (%)	95/181 (52.5%)	11/40 (27.5%)	0.010
Jaundice, n/N (%)	10/181 (5.5%)	0/40 (0.0%)	0.281
Duration of symptoms prior to surgery (days), mean ± SD	3.51 ± 3.47	3.24 ± 3.13	0.636
Time from admission to surgery (days), mean ± SD	1.24 ± 2.69	2.08 ± 2.90	0.100
Same-day surgery (0)	80/181 (44.2%)	13/40 (32.5%)	
Next-day surgery (1)	54/181 (29.8%)	11/40 (27.5%)	
Surgery ≥ 2 days after admission	47/181 (26.0%)	16/40 (40%)	

**Table 2:** Types of postoperative complications within 30 days after cholecystectomy. Values are presented as n/N (%). Categories are not mutually exclusive; individual patients could contribute to more than one complication category. Complications were grouped into clinically relevant categories based on a chart review of events occurring within 30 days after surgery.

Complications Category	ACC (*n*)	ACC (%)	AAC (*n*)	AAC (%)
Biliary complications	8	4.4	1	2.5
Intra-abdominal infections or fluid collections	6	3.3	1	2.5
Wound complications	8	4.4	2	5
Bleeding of hematologic complications	8	4.4	5	12.5
Cardiopulmonary complications	6	3.3	8	20
Renal or urinary complications	2	1.1	2	5
Gastrointestinal complications	5	2.8	1	2.5
Reinterventions or invasive management	4	2.2	3	7.5
Readmission	8	4.4	4	10
Death	10	5.5	13	32.5

**Table 3:** Baseline characteristics. Values are presents as mean ± SD, median [IQR], or n/N (%). Δ denotes mean difference between groups (acalculous − calculous). Abbreviations: ASA—American Society of Anesthesiologists; CI—confidence interval; IQR—interquartile range; OR—odds ratio; SD—standard deviation.

Variable	Calculous (*n* = 181)	Acalculous (*n* = 40)	Effect Size (95% CI)	*p*-Value
Sex (male), n/N (%)	85/181 (47.0%)	30/40 (75.0%)	OR 0.30 (0.12–0.67)	0.002
Age (years), mean ± SD	61.9 ± 16.0	72.2 ± 11.5	Δ 10.4 (6.1–14.7)	<0.001
Charlson Comorbidity Index, median [IQR]	3.0 [1.0–4.0]	5.0 [3.0–6.0]	—	<0.001
ASA Physical Status, n/N (%)	I: 30/181 (16.6%)	I: 0/40 (0.0%)	—	<0.001
	II: 78/181 (43.1%)	II: 3/40 (7.5%)		
	III: 52/181 (28.7%)	III: 23/40 (57.5%)		
	IV–V: 21/181 (11.6%)	IV–V: 14/40 (35.0%)		
Any blood thinner, n/N (%)	55/181 (30.4%)	26/40 (65%)	OR 0.24 (0.11–0.48)	<0.001

**Table 4:** Multivariable analyses of early postoperative outcomes after cholecystectomy for acute cholecystitis. The results of four prespecified adjusted models are presented. Models 1–3 (any postoperative complication, severe complications [Clavien–Dindo ≥ IIIa], and 30-day mortality) used binary logistic regression and are expressed as odds ratios (ORs) with 95% confidence intervals. Model 4 (Comprehensive Complication Index, CCI) used ordinary least-squares linear regression and is expressed as a regression coefficient (β) with a 95% confidence interval. Abbreviations: AAC—acute acalculous cholecystitis; ACC—acute calculous cholecystitis; ASA—American Society of Anesthesiologists Physical Status; CCI—Comprehensive Complication Index; CI—confidence interval; OR—odds ratio.

Variable	M1: Any Complication OR (95%CI)	M2: Severe Complication OR (95%CI)	M3: Mortality OR (95%CI)	M4: CCI β (95%CI)
AAC (vs. ACC)	2.76 (1.16–6.54)	2.30 (0.96–5.55)	4.08 (1.49–11.16)	+18.45 (7.93–28.98)
Age	1.03 (1.00–1.07)	1.05 (1.01–1.09)	1.07 (1.02–1.12)	+0.31 (−0.03–0.65)
Sex (M)	0.90 (0.44–1.84)	0.95 (0.44–2.05)	-	−3.89 (−11.22–3.44)
ASA	1.36 (0.82–2.25)	1.30 (0.76–2.24)	1.90 (0.97–3.71)	+2.67 (−2.64–7.99)
Charlson CI	1.12 (0.88–1.42)	1.10 (0.87–1.39)	-	+1.17 (−1.46–3.80)
Time to operation	1.05 (0.92–1.19)	0.99 (0.87–1.12)	-	+0.27 (−1.10–1.64)
Open (vs. laparoscopy)	1.67 (0.74–3.75)	-	-	+7.97 (−0.79–16.73)
Events (*n*)	66	44	23	221, R^2^ = 0.250

**Table 5:** Operative characteristics and postoperative outcomes. Values are presented as n/N (%) or mean ± SD. Δ denotes mean difference between groups (acalculous − calculous). Abbreviations: OR—odds ratio; SD—standard deviation; IQR—interquartile range.

Outcome	Calculous (*n* = 181)	Acalculous (*n* = 40)	Effect size (95% ci)	*p*-Value
Operative approach (Open/Laparoscopic/ Conversion)	**Open** 80/181 (44.2%);	**Open** 32/40 (80.0%);	—	<0.001
	**Lap** 71/181 (39.2%);	**Lap** 7/40 (17.5%);		
	**Conv** 30/181 (16.6%)	**Conv** 1/40 (2.5%)		
Intra-operative complications, n/N (%)	8/181 (4.4%)	5/40 (12.5%)	OR 0.33 (0.09–1.34)	0.064
30-day mortality, n/N (%)	10/181 (5.5%)	13/40 (32.5%)	OR 0.12 (0.04–0.34)	<0.001
30-day readmission, n/N (%)	8/181 (4.4%)	4/40 (10.0%)	OR 0.42 (0.11–2.00)	0.237
Length of stay post-op (days), median, [IQR]	3 [2–5]	4.5 [2–7]		0.213

## Data Availability

The original contributions presented in this study are included in the article. Further inquiries can be directed to the corresponding author.

## Supplementary Materials

The following supporting information can be downloaded at: https://www.mdpi.com/article/10.3390/medicina62050834/s1, Table S1: Characteristics of preoperative inflammatory markers.
