# Supplementary material for: Comparative Early Postoperative Outcomes in Acute Calculous vs. Acute Acalculous Cholecystitis: A Retrospective Analysis
Source: Medicina (Kaunas). 2026 Apr 27;62(5):834. doi: 10.3390/medicina62050834 (PMC13208797; doi:10.3390/medicina62050834)
Supplement: Supplementary file 1 [file medicina-62-00834-s001.zip › medicina-4217883-supplementary.pdf]

Table S1. Characteristics of preoperative inflammatory markers. Values are presented as median [IQR]. Adjusted p-values were calculated using the Benjamini–Hochberg procedure across the eight biomarker comparisons. **Abbreviations.** IQR, interquartile range.

Timepoint	Marker (unit)	Calculous (median [IQR])	Acalculous (median [IQR])	p-value (adjusted)
Admission	CRP (mg/L)	86.6 [15.6–172.3]	180.7 [56.3–258.9]	0.006
Day of surgery	CRP (mg/L)	134.0 [62.2–224.4]	182.8 [124.9–289.5]	0.022
Admission	NLR	7.5 [4.4–13.2]	14.1 [6.6–24.0]	0.004
Day of surgery	NLR	7.5 [4.4–12.4]	17.9 [9.5–31.9]	<0.001
Admission	WBC (G/L)	12.3 [9.1–17.1]	13.5 [10.0–17.4]	0.156
Day of surgery	WBC (G/L)	12.3 [9.2–15.9]	13.9 [9.9–20.5]	0.071
Admission	PLR	185.3 [131.9–260.9]	225.0 [135.2–370.7]	0.156
Day of surgery	PLR	178.9 [127.5–247.9]	252.1 [146.9–483.0]	0.013
